# An empirical study of preprocessing techniques with convolutional neural networks for accurate detection of chronic ocular diseases using fundus images

**DOI:** 10.1007/s10489-022-03490-8

**Published:** 2022-04-30

**Authors:** Veena Mayya, Sowmya Kamath S, Uma Kulkarni, Divyalakshmi Kaiyoor Surya, U Rajendra Acharya

**Affiliations:** 1 Healthcare Analytics and Language Engineering (HALE) Lab, Department of Information Technology, National Institute of Technology Karnataka, Surathkal, Mangalore 575025 India; 2 Department of Information, Communication Technology, Manipal Institute of Technology, Manipal Academy of Higher Education (MAHE), Manipal, 576104 Karnataka India; 3 Department of Ophthalmology, Yenepoya Medical College, Yenepoya (Deemed to be) University, Mangalore, 575018 India; 4 School of Engineering, Ngee Ann Polytechnic, Clementi, 599489 Singapore; 5 Department of Biomedical Engineering, School of Science and Technology, Singapore University of Social Sciences, Singapore, S599494 Singapore; 6 Department of Biomedical Informatics and Medical Engineering, Asia University, Taichung, 41354 Taiwan

**Keywords:** Healthcare informatics, Clinical decision support systems, Explainability, Fundus imaging, Convolutional neural networks

## Abstract

Chronic Ocular Diseases (COD) such as myopia, diabetic retinopathy, age-related macular degeneration, glaucoma, and cataract can affect the eye and may even lead to severe vision impairment or blindness. According to a recent World Health Organization (WHO) report on vision, at least 2.2 billion individuals worldwide suffer from vision impairment. Often, overt signs indicative of COD do not manifest until the disease has progressed to an advanced stage. However, if COD is detected early, vision impairment can be avoided by early intervention and cost-effective treatment. Ophthalmologists are trained to detect COD by examining certain minute changes in the retina, such as microaneurysms, macular edema, hemorrhages, and alterations in the blood vessels. The range of eye conditions is diverse, and each of these conditions requires a unique patient-specific treatment. Convolutional neural networks (CNNs) have demonstrated significant potential in multi-disciplinary fields, including the detection of a variety of eye diseases. In this study, we combined several preprocessing approaches with convolutional neural networks to accurately detect COD in eye fundus images. To the best of our knowledge, this is the first work that provides a qualitative analysis of preprocessing approaches for COD classification using CNN models. Experimental results demonstrate that CNNs trained on the region of interest segmented images outperform the models trained on the original input images by a substantial margin. Additionally, an ensemble of three preprocessing techniques outperformed other state-of-the-art approaches by 30% and 3%, in terms of Kappa and *F*_1_ scores, respectively. The developed prototype has been extensively tested and can be evaluated on more comprehensive COD datasets for deployment in the clinical setup.

## Introduction

According to WHO projections [1], the global population suffering from myopia will reach 3.36 billion by 2030, while those suffering from age-related macular degeneration (AMD), glaucoma, and diabetic retinopathy (DR) will reach 243.3 million, 95.4 million, and 180.6 million, respectively. Early detection of COD is essential for clinical decision-making and can significantly reduce the risk of vision impairment. Regular screening is an important step toward early detection. However, the process adopted for screening is primarily a manual investigation [2, 3]. This makes it impractical to scale, given the wide range of diseases and ever-growing patient population. Additionally, the doctor-to-patient ratio is lower in most of the third world. Though over two lakh expert ophthalmologists are practising globally, there is a severe shortage of ophthalmologists in underdeveloped countries [4]. In underdeveloped nations, the number of expert ophthalmologists per million people is reported to be just 11 [4], which is highly inadequate when compared to the growing COD patient population. Automated systems can aid in the early detection of COD via tele-ophthalmology in rural areas where there is a shortage of retina specialists. Given the ever-increasing patient population every day, manual screening is highly time-consuming, and the treatment capacity is often limited due to low doctor:patient ratios across the world. It is crucial to develop intelligent computational systems like multimodal image retrieval [5–7] and clinical decision support systems (CDSSs) [8–10] that accommodate these needs by facilitating automated diagnostic image management for early detection of chronic diseases at the patient level. The detection of ocular diseases can be considered as a multi-label classification problem involving the binary classification of multiple diagnostic labels. Labels are assigned to identify a specific diagnostic condition recorded as a binary indicator, considering the patient’s eye conditions. Recently, there has been significant interest in developing an automated COD screening system capable of detecting various eye disorders. These models use colored fundus photographs, fluorescein angiography, optical coherence tomography (OCT), optical coherence tomography angiography (OCTA), and other ocular imaging data. Most earlier computer-aided screening methods used digital image-processing based techniques (IPT) [11]. Later, supervised machine learning techniques (MLT) were developed, which extract features using predefined rules or statistical and structural metrics [12–14]. Over the years, research directions have shifted towards end-to-end, intelligent predictive systems that use the predictive power of deep neural networks, owing to their data-driven-feature learning capabilities. Deep neural networks have achieved state-of-the-art performance for various clinical prediction and diagnostic tasks over multiple patient data modalities [15, 16], lifestyle diseases [17–20] and CODs [21–25].

Convolutional neural networks (CNN) have shown promising performance in detecting COD like glaucoma, DR, and AMD, using color fundoscopy images [24, 26–29]. Although several preprocessing techniques are used to detect COD using CNN, a comprehensive experimental study on the effect of preprocessing on the performance of CNN is yet to be explored. In this context, we design and conduct a series of experiments comparing the COD detection performances of CNN architectures, exploring possible alterations in preprocessing and augmentation methods that can enable existing CNN models to distinctly focus and learn the relevant features from minute ocular lesions. This study also aims to delve deep into effective preprocessing techniques that can boost the patient-level predictive performance of DL-based diagnostic systems. Additionally, a pilot study was carried out to understand the efficient preprocessing techniques that can aid ophthalmologists in clinical decisions. The key contributions of this study can be summarized as follows: 1) Development of a region of interest (RoI) detection algorithm for precisely segmenting the fundus region for efficient CNN training in learning minute lesions. 2) Provide a comprehensive comparative COD classification performance evaluation of state-of-the-art DL architectures 3) Present the findings of a wide range of experiments that document the effects of preprocessing, data augmentation, and ensemble methods. 4) Conduct a pilot study to understand the effective preprocessing methods that can aid ophthalmologists in their clinical decisions.

The rest of the paper is organized as follows: Section 2 provides a comprehensive review of the most relevant and effective DL-based COD detection methods reported in the literature. Different preprocessing methods and DL-based COD diagnostic systems have also been reviewed. Section 3 details the preprocessing, data augmentation, and DL architectures used to analyze the performance of the COD detection system. Section 4 documents the evaluation of the DL models and details the extensive experiments conducted on DL models trained under different preprocessing, augmentation, and ensemble methods. Section 5 summarizes the proposed experimental study and presents future work.

## Review of existing works

Color fundus images captured from fundus cameras are used for the early diagnosis of COD. The transformations on the input color fundus images before feeding them to the deep neural models play a crucial role in improving the diagnostic performance. Preprocessing reduces the possible noise in color fundus images such as irregular illumination, low contrast, unimportant features, etc., thus improving the performance of DL-based COD diagnosis. A few digital image preprocessing techniques reported in the literature are adaptable to all COD (hereby referred to as generic techniques). On the other hand, a few preprocessing methods are only versatile for particular ocular diseases like DR, glaucoma, cataract, or AMD (hereby referred to as specific preprocessing techniques).

The most common clinical signs based on which ophthalmologists identify the progression of DR in the fundus images are microaneurysms, haemorrhages, exudates, thickening within one disc diameter from the foveal centre, and retinal neovascularisation [30, 31]. Thus, the lesion regions are segmented for building DL-based automated DR diagnostic systems. Chalakkal et al. [32] investigated the effect of fovea segmentation on macular oedema screening using DL-based transfer learning approaches. The authors critically analysed the effects of limiting the RoI to the fovea and reported improved performance due to RoI segmentation than considering the entire fundus image. The progressive alterations in the retinal vessels are also crucial for identifying DR [33]. Several researchers [34–37] have segmented the retinal vessel structure from the input color fundus images before feeding them to the DL-based diagnostic system.

Damage to the optic nerve is the primary cause of vision loss in glaucoma. Glaucoma can be detected by examining the abnormalities of the optic disc. Several works [38–44] employed cropping/segmentation of optic disc regions, and then used CNN to diagnose glaucoma using color fundus images. Juneja et al. [43] proposed a modified version of U-net (G-net) to segment the optic disc and cup region, after which they used the ratio of these areas to predict glaucoma. Zhao et al [41] adapted a template matching method for locating and cropping the bounding region around the optic disc and proposed MFPPNet to screen glaucoma. Modified U-Net [45] is predominantly used for optic disc segmentation [39, 40, 44] and then transfer learning is applied to the cropped region to screen glaucoma. Pathan et al. [13] used inpainting to eliminate the vascular structure before segmenting the optic disc/cup regions. A recent study, [46, 47] suggests that various regions, like the arterioles, venules, etc., are also associated with high-tension open-angle glaucoma. The performance of CNN using only optic disc cropped regions has been experimented with, a systematic experimental evaluation of other preprocessing methods in CNN has yet to be undertaken.

The increase in protein aggregation in the lens does not allow light to pass through the lens and may lead to cataract. Xu et al. [48] divided the input fundus images into eight local patches based on ophthalmologists’ recommendations for automatic cataract grading using CNN. Using the unified rectangular fundus images, Zhang et al. [49] extracted the high-level texture features using a CNN model and supervised SVM to grade the severity of the cataract. Imran et al. [50] extracted the image green channel, resized the images, and used CNNs for feature extraction and SVM for cataract severity identification. Thus, there is a wide scope for the experimental evaluation of other preprocessing methods using CNN for automatic cataract diagnosis.

Though the choice of preprocessing techniques is dependent on the type and requirements of a particular ocular disease diagnosis, a thorough analysis of the effect of preprocessing on the efficiency of DL models has not been undertaken, to the best of our knowledge. The qualitative analysis of the adapted preprocessing strategies is not well discussed in the existing literature. Preprocessing of input fundus images necessitates the use of computational resources. A few preprocessing methods may improve the predictive performance, while others may have the opposite impact. Some preprocessing techniques may be best suited for specific ocular diseases. Still, they may not meet the clinical needs in real-time, particularly in scenarios where there is a lot of variation in eye diseases. Thus, there is significant scope for conducting a comprehensive, systematic assessment of such techniques’ relative strengths and weaknesses for quantifying their usefulness in automated chronic disease diagnosis.

CNN based models have been recently used for COD detection by many researchers. Islam et al. [51] proposed a shallow CNN to predict CLAHE preprocessed RGB fundus images with the dimension of (32 × 32). With such tiny image dimensions, the most relevant information is lost, resulting in neural network overfitting. Wang et al. [52] applied histogram equalization on both (448 × 448) RGB and grayscale images. Then, two EfficientNet-B3 [53] networks were separately trained on these images, and the predicted values for networks were averaged to get the final prediction. The increased input image dimensions necessitate the use of more training parameters, which increases the computing resources required during both the training and inference phases. Gour and Khanna [54] concatenated left and right fundus RGB images of dimension (256 × 256) and classified COD using sigmoid activation function. Li et al. [55] fused the left and right eye CNN network features and classified COD using eight separate classifiers. He et al. [25, 56] extracted ResNet features of left and right fundus RGB images of dimension (448 × 448) and refined the features using spatial correlation module. The authors randomly split the training ODIR dataset and cross-validated the proposed method on 1166 fundus images. He et al. [57] improved prior efforts [25, 56] by training a teacher network on fused features from both eye images and the 102 diagnostic keywords. While it is not always prudent to get fundus images of both eyes at the same visit, the developed CDSS should be adaptable to such circumstances. Due to the fusion of multiple input fundus images, visualizing the information learned by the CNN models for predicting the output COD is a challenging task. For CDSS to be adaptable in real-world circumstances, providing a transparent, explainable decision (even if it is wrong) is considerably more acceptable than putting forth a highly accurate, non-transparent decision, primarily due to the trust barrier between ophthalmologists and automated systems. To address these issues, we propose an automated RoI segmentation and ensemble technique that enables CDSSs to learn minute lesions for accurate early COD detection and allows visualization of the input image features that contributed to it.

## Methodology

**Fig. 1:**
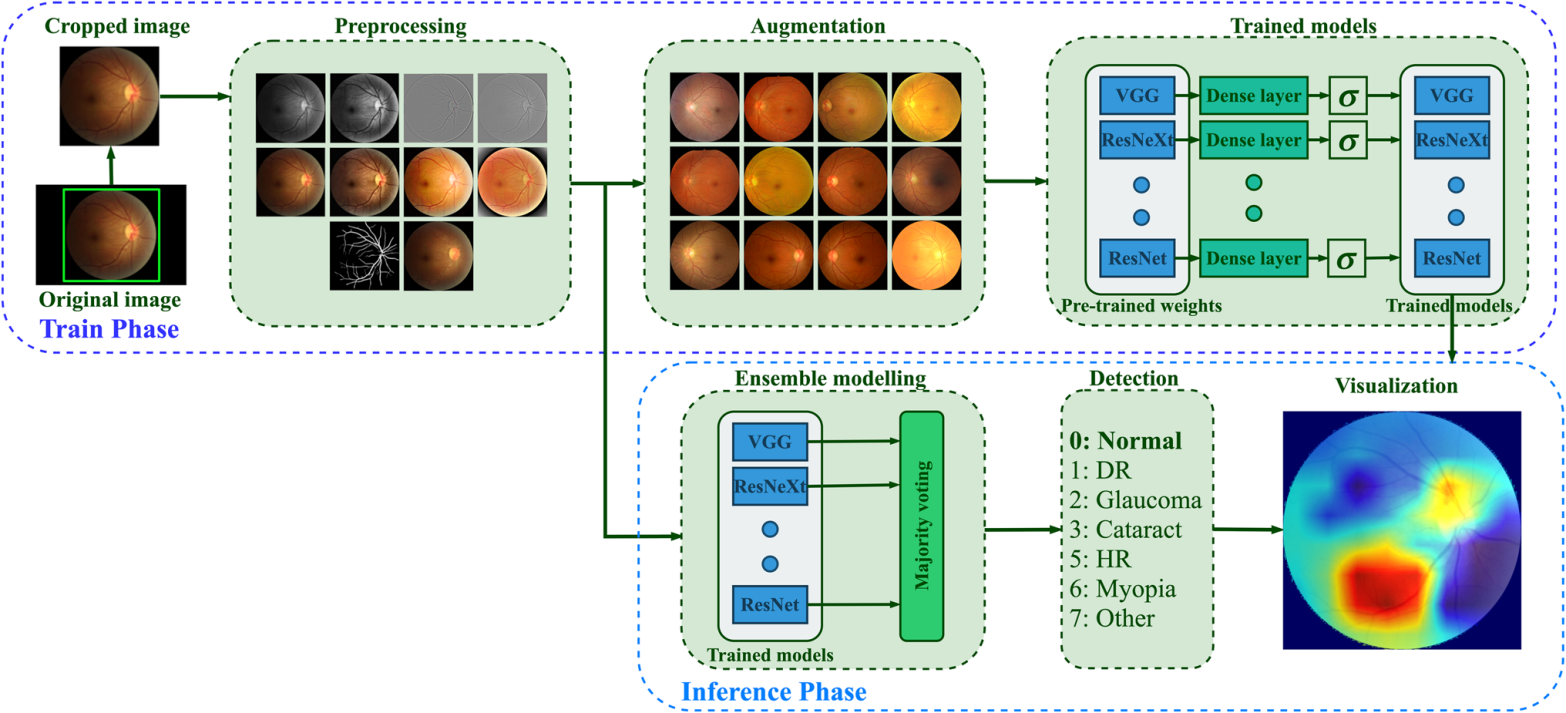
Overall methodology employed in the proposed study for COD classification

The role of preprocessing in improving the performance of the DL-based diagnostic system is investigated in this study by experimenting with two specific (vessel segmentation and inpainting) and nine generic preprocessing techniques. Figure 1 provides an overview of the training methodology, and further details regarding the preprocessing and augmentation methods used in the course of experiments have been elaborated below.

### Automatic region of interest segmentation

The gradient Hough transform is used to segment the foreground circular fundus region. The parameters are chosen based on the validation of 500 fundus images. The inverse of the ratio of accumulator resolution to image resolution is set to 1, and the minimum distance between the detected circles’ centre coordinates is set to 20. The accumulator threshold value is set to 30, and the gradient value for edge detection is set to 50. The minimum radius (in pixels) is set to 1/4 of the input dimensions’ minimum, while the maximum size is set to the input dimensions’ maximum size. The bounding box is formed for the detected circular region, ensuring that the cropped region is within the image dimension. The appropriate circular area (from the detected circles) is chosen, ensuring that non-zero pixels outside the cropped region are within five rows and columns.

**Fig. 2:**
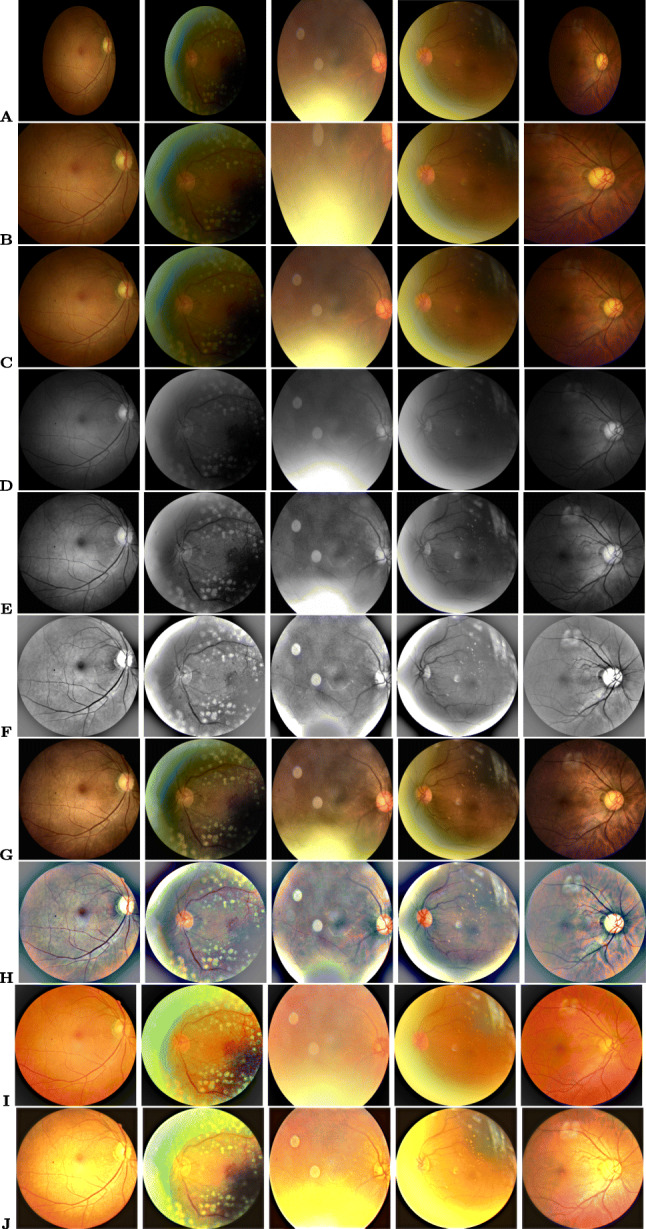
Results of preprocessing on input sample images. **A**)Original, **B**)Otsu’s thresholding segmentation, **C**)Proposed Hough transform segmentation, **D**)Green channel, **E**)Green channel CLAHE, **F**)Green channel Gaussian convolution, **G**)RGB CLAHE, **H**)RGB Gaussian convolution, **I**)MSR, and **J**)MIRNET images

The automatic foreground cropping mechanism is described in detail in Algorithm 1. A few sample input fundus images and the corresponding foreground segments obtained after processing are shown in Fig. 2c. We also experimented with Otsu thresholding [58] using the largest contour crop method, but the foreground regions obtained were clipped in the darker images. With our proposed automatic segmentation approach, only the background regions are cropped without losing useful details (refer Fig. 2(a-c)). 
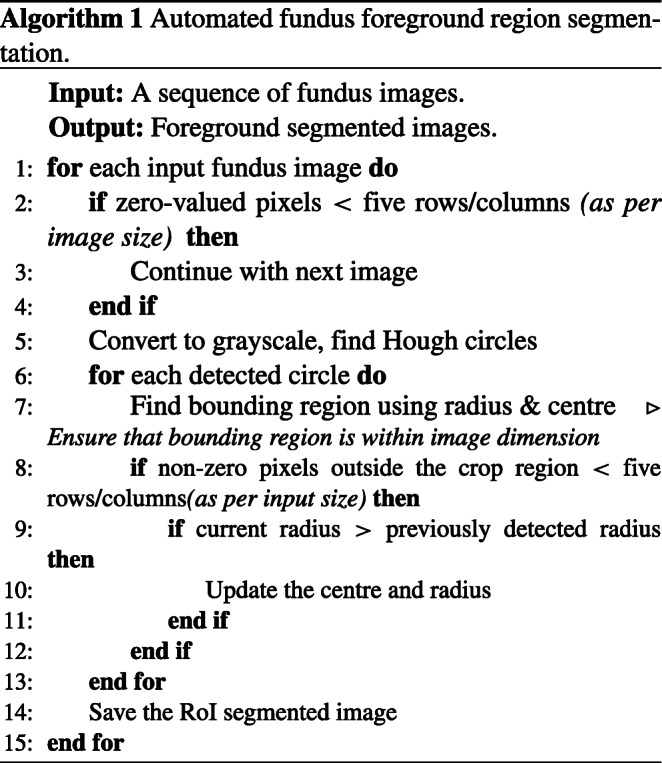

### Image enhancement

During this process, we aim to enhance the visual quality of the fundus images for improved learning performance. To improve the visual quality of input fundus images, experiments are carried out using CLAHE [59] (on both green and RGB channels), Gaussian filter convolution, Multiscale Retinex (MSR) [60], and multiscale residual block network (MIRNET) [61] approaches. The CLAHE enhanced image is obtained by applying the normalized intensity histograms *P*_*n*_ (see (1)) to the image patches (5 × 5), which are defined as a matrix M (*r* × *c*) with values (pixel intensity) ranging from 0 to *L* − 1. The resulting image patch *I*_*eq*_ is defined by (2). The contrast factor (or clip limit) that limits the slope associated with the gray-level assignment scheme in CLAHE is set to 2.

1 $$ \text{\textit{P}}_{n} = \frac{\text{Number of pixels with intensity n}}{\text{Total number of pixels}} \hspace {10 pt}n = 0,1 {\dots} L-1  $$

2 $$ \text{\textit{I}}_{eq} = \lfloor{(L-1)\sum\limits_{n=0}^{M_{i,j}}P_{n}}\rfloor  $$

In this work, a Gaussian filter convolved (*blurred*) image is blended with the original image to improve image contrast. The resulting enhanced image *I*_*gs*_ is defined by (3). *G*(*h*,*w*) represents a Gaussian filter with a scale *σ* and ∗ the convolution operator. The parameter values are determined based on experimentation and are set as *α* = 4, *β* = − 4, *σ* = 10, and *γ* = 128. The MSR algorithm [62, 63] is adapted to improve the local contrast enhancement of the fundus images. MSR is implemented by [60], based on the Retinex [64] theory, which attempts to model human visual color perception. MSR improves the local contrast of the fundus image as per the desired scales (*sigma*_*n*_) of the Gaussian kernels (refer (4)). *N* is set to three scales ([5,35,150]) in this study, and the weight factor associated with the Gaussian function (*W*_*n*_) is set to 1/3.

3 $$ \text{\textit{I}}_{gs}(h,w) = \alpha I_{orig}(h,w) + \beta G(h,w,\sigma) * I_{orig}(h,w)+ \gamma  $$

4 $$ \begin{array}{@{}rcl@{}} I(h,w)_{ms-retinex} &=& \sum\limits^{N}W_{n} (log(I(h,w))\\ &&-log((I(h,w)) * G(h,w,\sigma_{n})) )  \end{array} $$

Recently, [61] proposed the MIRNET model that integrates parallel multi-resolution convolution, spatial and channel attention for image enhancement. The pre-trained weights of this model are utilized in our work to enhance the contrast of fundus images. From Fig. 2, the effect of each preprocessing phase considered as part of our experiments on sample input fundus images can be observed. As can be seen from Fig. 2, MSR increases the brightness while maintaining/improving the overall visual quality. CLAHE and MIRNET enhance the visual quality of the image but also introduce additional artefacts, while the green channel retains the original image’s visual quality.

### Vessel segmentation

**Fig. 3:**
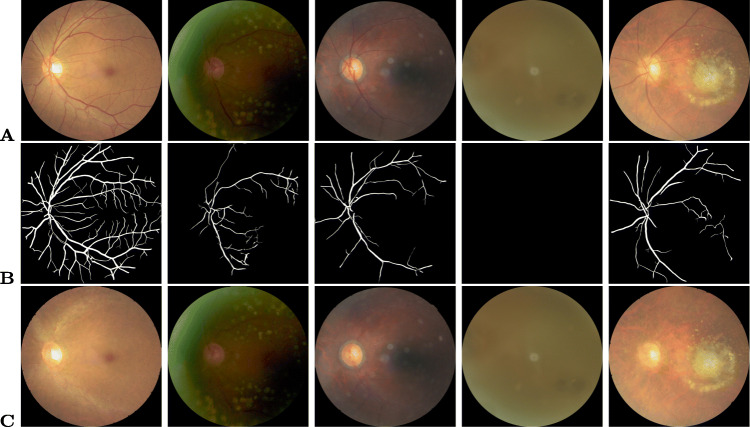
Results of segmentation and inpainting of vessel structure on sample input images. **A**)Original (normal, DR, glaucoma, cataract and AMD), **B**)Vessel segmentation, **C**)Vessel inpainting images

The progressive changes of retinal vessels are crucial for detecting COD, for which preprocessing is performed through the segmentation of blood vessel regions. The DRIVE dataset [65] is used to train the RetinaNet [66] model for this purpose. CLAHE is applied to increase the contrast of the color fundus images, and the generative neural model [66] is re-trained for the segmentation of vessel masks. Regions with intensities less than the threshold (20) are set to zero in the mask. If the number of connected pixels in each connected component is less than 100, the intensities are set to zero. Algorithm 2 details the steps involved in vessel segmentation. To observe the impact of vessel structure on the COD detection performance, a process of background color inpainting is applied to the segmented vessel masks, using a pre-trained generative model [67]. Figure 3 shows some sample images from various COD classes. 
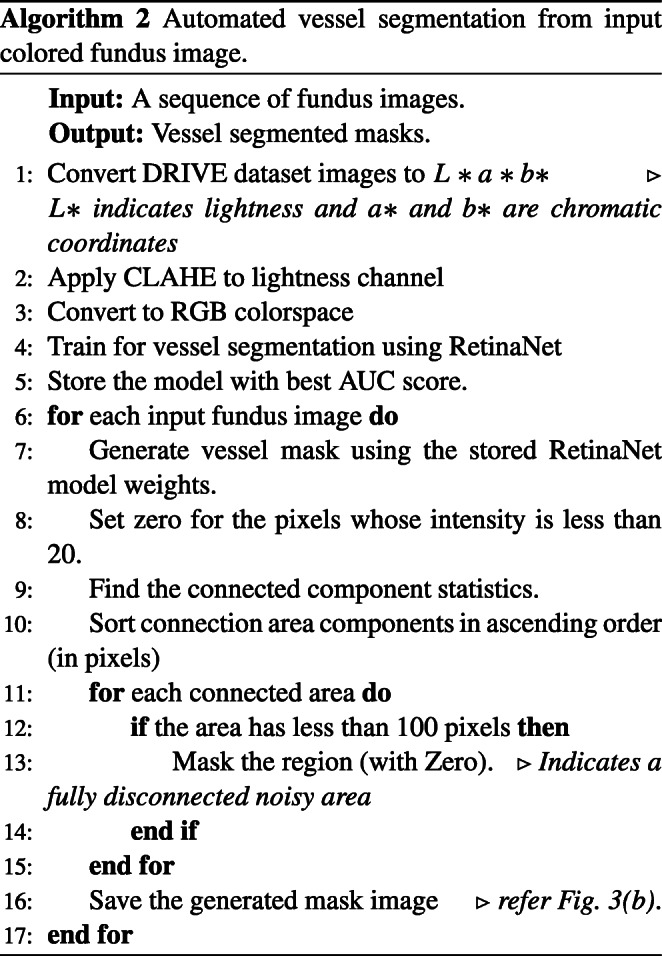

### Data augmentation

**Fig. 4:**
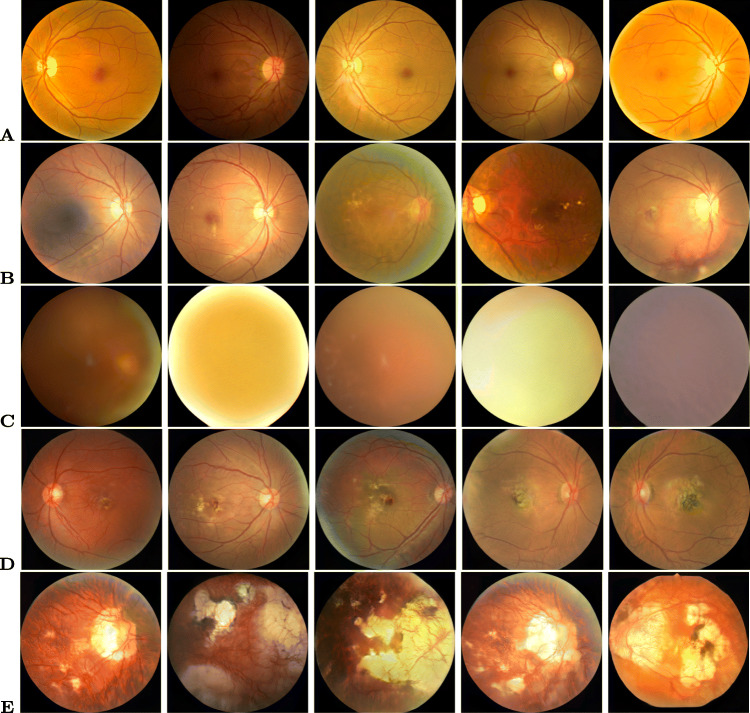
Sample of generated fundus images using StyleGAN2 for the given ocular conditions. **A**)Normal, **B**)DR, **C**)Cataract, **D**)AMD, **E**)Myopia

For effective learning performance, generalizable deep neural models require a large number of labelled images. A variety of data augmentation techniques are widely used to deal with the limited number of training images. In our work, batch-level and condition-level data augmentation techniques are incorporated to increase the number of images used to train the neural models. In batch-level augmentation, horizontal and vertical flipped images, as well as random angle rotated images, are generated and added to the training set. In condition-level augmentation, training images are augmented conditionally using the StyleGAN2 model [68]. The StyleGAN2 is applied to the training dataset, using the textual descriptions of the ocular diseases. The diseases with fewer than five training images were excluded, which resulted in 48 conditions. The network has been trained for 25 million training images. Figure 4 presents a representative sample of fundus images generated for the given COD. As a result of the data augmentation, over 21,000 training images were obtained by augmenting 300 images for each ocular condition.

### Convolutional neural models

CNNs have shown exceptional performance in various computer vision tasks, including the classification of COD. We base our experiments on eleven prominent CNN models that have demonstrated high performance on the ImageNet [69] challenge dataset. We experimented with SqueezeNet [70], MobileNet [71], Inception [72, 73], DenseNet [74], EfficientNet [53], ResNeXt [75], ResNet [76], WideResNet [77], and VGG16 [78], using both cropped and non-cropped fundus images. Table 1 lists the number of training parameters used for the DL models in ascending order. The model parameters are initialized using the ImageNet pre-trained weights. The final dense layer is initialized with the same random seed for all the models. This ensures that all models are trained as per a common parameter setting. After observing the outcomes of the experiments, the model with the highest *F*_1_ score is utilized as a baseline for examining the effect of various preprocessing techniques on CNN performance. Fundus images are preprocessed individually and fed into the CNN models for inference. The maximum score is regarded as the final screening result at the patient level.

**Table 1:** Details of model training parameters

Model	Parameters *(in millions)*
SqueezeNet [70]	0.726600
MobileNetv2 [71],	2.234120
Inceptionv1 [72]	5.608104
DenseNet121 [74]	6.962056
EfficientNet-B3 [53],	10.708528
ResNeXt50 [75]	22.996296
ResNet50 [76],	23.524424
Inceptionv3 [73]	25.128656
EfficientNet-B7 [53]	63.807448
WideResNet50 [77]	66.850632
VGG16 [78]	134.29332

COD classification is a multi-label classification problem that requires binary classification of multiple diagnostic labels, each label indicating a specific COD. As a result, in this study, binary predictions are used as target scores, with actual and predicted values compared pairwise. A multi-label one-versus-all loss based on max-entropy is used to train the CNN, as illustrated in (5) (where *i*∈ $\{0 \dots , N \}$, *y*[*i*] ∈ {0,1}). In this study, *Y* represents the actual target labels, while $\hat {y}$ represents the predicted labels of dimension (*N*,*C*) (where *N* is the batch size and *C* is the number of classes= 8).

5 $$ \begin{array}{@{}rcl@{}} \text{loss}(\hat{y},y) &=& - \frac{1}{C} * \sum\limits_{i} {y[i] * log((1+exp(-\hat{y}[i]))^{-1})}\\ &&+ (1-y[i]) * log\left( \frac{exp(-\hat{y}[i])}{(1+exp(-\hat{y}[i]))}\right) \end{array} $$

## Experimental results and discussion

For the experimental evaluation of the proposed approaches, the ODIR-5K (Ocular Disease Intelligent Recognition) challenge dataset [79], consisting of a total of 5,000 patients’ data, was employed. The training dataset comprises 3,500 patient records with 7,000 fundus images, and the final label is based on both eye conditions. The dataset is highly imbalanced. It contains over 3,000 normal images but only 190 hypertensive retinopathy images. About 500 images have multiple labels, and the “*Others*” category consists of more than 20 distinct eye diseases. The statistics of the training data are summarized in Table 2. Automatic relabeling was carried out based on the textual information available for each eye condition, and the validity was checked by comparing the union of the labels with the final available labels. For example: the image *0_left.jpg* was labelled *00010000*, as *cataract* was present in the textual description. The *0_right.jpg* was labelled *10000000* as the text included *normal fundus*. The union *00010000* was verified with the final available label *00010000*. Normal labels are considered only when both eyes have the text *normal fundus*, ignored otherwise. Some representative fundus images along with the final available labels are listed in Table 3. All the images are resized to 256 × 256.

**Table 2:** Details of ODIR training data

Type of COD (Class)	Training images
Normal	3,098
DR	1,801
Others	1,200
Glaucoma	326
Cataract	313
AMD	280
Myopia	268
Hypertension	193

**Table 3:** Sample fundoscopy images from ODIR dataset

Final label	Right & left eye images	
DR		
AMD + DR		
DR + Myopia + Others		
DR + Cataract + Others		

### Evaluation metrics

Several standard metrics were used for validation purpose. *F*_1_ score is used as a primary metric for validating the output of preprocessing techniques, as it is a weighted harmonic mean of precision and recall (see (6)). Thus, models with higher *F*_1_ scores are expected to improve the system’s predictability. The *F*_1_ metric is also more indicative than the standard accuracy score because it accounts for true and false positives (TP and FP) as well as true and false negatives (TN and FN). The precision and recall for the neural system over *C* classes are computed using (7) and are macro-averaged over the target output classes. In addition to these metrics, we also report the models’ performance using the area under the curve (AUC) and Kappa score (average of Cohen’s kappa for each label) multi-label classification metrics. The area under the ROC curve is referred to as the AUC. The model’s classification accuracy improves as it gets closer to 1. It is often used to determine the model’s stability. Cohen’s Kappa [80] (see 8) is a quantitative measure of reliability - a score of 0 indicates that there is a random match, while a score of 1 means that the true and predicted labels are fully in the agreement.

6 $$  F_{\beta = 1} = (1 + \beta^{2}) \cdot \frac{\text{precision} \cdot \text{recall}}{(\beta^{2} \cdot \text{precision}) + \text{recall}} $$

7 $$  \text{precision} = \frac{1}{C}\text{ }\sum\limits_{c=1}^{C} \frac{\text{TP}_{c}}{\text{TP}_{c} + \text{FP}_{c}}; \text{recall} = \frac{1}{C}\sum\limits_{c=1}^{C} \frac{\text{TP}_{c}}{\text{TP}_{c} + \text{FN}_{c}} $$

8 $$ Kappa_{score} = \frac{P_{o}-P_{e}}{1-Pe};  $$

### Observed results

During inference, the left and right eye images are both considered, and the final ocular condition is determined by a label-wise maximum score among two output predictions. The ODIR test set contains 500 unlabeled patient records (1,000 images) that can be labelled in any of the eight possible ways. Thus, if a single label is correctly predicted, an *F*_1_ score of 0.00025 is achieved, demonstrating the significance of prediction scores. Table 4 summarizes the results obtained with original color and cropped images obtained using state-of-the-art neural models with test ODIR data.^1^ The experimentation is conducted using a batch-level augmentation method (please refer Section 3.4). The performance of the top scoring neural model (ResNeXt50) with RoI crop is benchmarked using several preprocessing methods. As stated earlier, nine preprocessing methods have been employed, and the results are shown in Table 5. Experimentation was carried out to evaluate the top three performing neural models and further analyze the top-performing prepossessing techniques. The results are shown in Table 6. It can be observed that results are comparable to ResNeXt50 and the best results are obtained with RGB RoI cropped images.

**Table 4:** Observed performance for state-of-the-art DL models on the testset

	With original image			With cropped image		
	Kappa	AUC	**F**_1_	Kappa	AUC	**F**_1_
DenseNet [74]	0.4659	0.7888	0.8698	0.5195	0.8210	0.8804
EfficientNetB3 [53]	0.4691	0.7922	0.8695	0.5090	0.8199	0.8803
EfficientNetB7 [53]	0.4677	0.7265	0.872	0.5260	0.8317	0.8845
Inceptionv1 [72]	0.4124	0.7746	0.8553	0.4733	0.8021	0.8718
Inceptionv3 [73]	0.3131	0.7201	0.8365	0.3882	0.7671	0.8535
MobileNet [71]	0.4664	0.7984	0.8703	0.4852	0.8191	0.8740
ResNet50 [76]	0.4110	0.7602	0.8575	0.5022	0.8093	0.8782
ResNeXt50 [75]	0.4733	0.7921	0.8717	0.5680	0.8606	0.8953
WideResNet [77]	0.4222	0.7634	0.8618	0.4734	0.8343	0.8743
SqueezeNet [70]	0.1347	0.6178	0.7838	0.1594	0.6412	0.7873
VGG16 [78]	0.5092	0.7828	0.8790	0.5268	0.8248	0.8813

**Table 5:** Results of ResNeXt50 (best performing model) with proposed preprocessing pipeline

Preprocessing method	Observed performance		
	Kappa	AUC	**F**_1_
Original image	0.4733	0.7921	0.8717
Cropped image	0.5680	0.8606	0.8953
Green channel	0.5336	0.8186	0.8882
Green channel+CLAHE	0.5198	0.8189	0.8840
Green channel+Gaussian	0.4898	0.8082	0.8777
RGB+CLAHE	0.5308	0.8368	0.8860
RGB+Gaussian	0.5260	0.8206	0.8840
Multiscale Retinex (MSR)	0.5330	0.8418	0.8865
MIRNET	0.4438	0.8403	0.8550
Vessel segmentation	0.3097	0.7212	0.8325
Vessel inpaint	0.4865	0.8500	0.8765

**Table 6:** Observations w.r.t top three performing models, when used with RoI cropped images

DL model	RGB	Green	MSR
ResNeXt50 [75]	0.8953	0.8882	0.8865
VGG16 [78]	0.8813	0.8498	0.8465
EfficientNetB7 [53]	0.8845	0.8668	0.8605

The ResNeXt50 model achieved the best *F*_1_, Kappa and AUC scores on the cropped images obtained using the proposed RoI segmentation algorithm. As per the strategy adopted for benchmarking experiments (discussed in Section 3.4), the ResNeXt50 model is trained on the augmented training set, using the proposed preprocessing pipeline for COD classification. The observed results are tabulated in Table 7. It can be observed that batch-level augmentation achieved the best performance. Therefore, for the rest of the experiments, it was utilized for training the models. The majority rule voting approach is used to ensemble the predictions of the top three DL models (ResNeXt50, EfficientNetB7 and VGG16) with only RoI cropped color images and the top three preprocessing approaches (RoI cropped, green channel, and MSR) trained with the ResNeXt50 model. The results of ensemble models’ performance are shown in Table 7 and the results of benchmarking experiments with respect to state-of-the-art DL models are summarized in Table 8.

**Table 7:** Comparative performance of proposed augmentation and ensemble techniques

Method	Observed performance		
	Kappa	AUC	**F**_1_
No augmentation	0.5246	0.8323	0.850
Batch-level *(Flip + Rotation)*	0.5680	0.8606	0.8953
Condition-level *(GAN)*	0.4228	0.8534	0.8710
Ensemble 1 *(ResNeXt50, EfficientNetB7 & VGG16)*	0.5815	0.8532	0.9008
Ensemble 2 *(RoI cropped, green channel & MSR)*	0.6081	0.8806	0.9070

**Table 8:** Comparative performance of proposed approaches against state-of-the-art techniques

No.	Models	Dataset	Observed performance		
			Kappa	AUC	**F**_1_
1	ResNet-101 backbone [25, 56]	1166 patients data (ODIR train set)	0.6370	0.9300	0.9130
2	ResNet-101 + Textual features [57]	1166 patients data (ODIR train set)	0.6410	0.9380	0.9130
3	Graph convolutional network [81]	996 images of 498 patients (ODIR)	0.5765	0.7816	0.8966
4	EfficientNet-B3 [52]	ODIR offline challenge test set	0.5200	0.7400	0.8900
5	Shallow CNN [51]	ODIR offline challenge test set	0.3100	0.8050	–
6	Two input VGG16 [54]	ODIR offline challenge test set	–	0.6888	0.8557
7	VGG-16 [55]	ODIR offline challenge test set	0.4494	0.8881	0.8730
8	Proposed pipeline (§ 3.1 +§ 3.4 +§ 3.5) with ResNeXt50	ODIR offline challenge test set	0.5680	0.8606	0.8953
9	Proposed DL ensemble (§ 3.1 +§ 3.4 +§ 3.5 +§ 4.3 )	ODIR offline challenge test set	0.5891	0.8610	0.9025
10	Proposed preprocessing ensemble (§ 3.1 + § 3.2 +§ 3.4 +§ 3.5 +§ 4.3)	ODIR offline challenge test set	0.6081	0.8806	0.9070

### Discussion

**Fig. 5:**
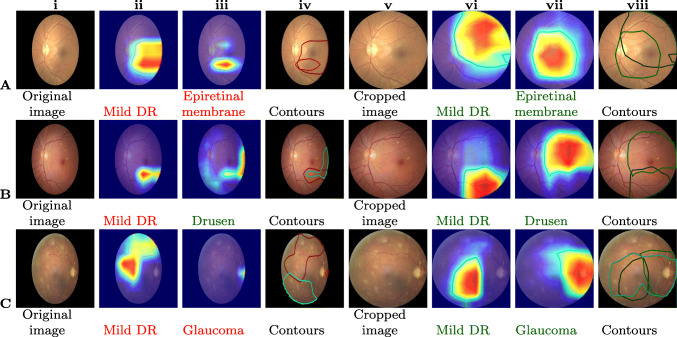
Visualization of Grad-CAM heatmap on the original input images. Columns **i-iv** show the original images, where as, columns **v-viii** are cropped versions. The annotated labels are **A**) Mild DR (D), epiretinal membrane (O); **B**) Mild DR (D), drusen (O); and **C**) Mild DR (D), glaucoma (G), vitreous degeneration (O)

During the extensive experiments conducted to evaluate the effectiveness of the proposed approaches, we observed that the models trained using cropped fundus images always outperformed those trained on non-cropped images by an average percentage difference of 15% Kappa score as shown in Table 4. This can be attributed to the enhanced predictability afforded due to the proposed RoI segmentation algorithm. To visualize the dominant features learned by the proposed model to detect a particular type of COD, we used Gradient-weighted class activation mapping (Grad-CAM) [82]. Figure 5 shows the Grad-CAM visualization for the original (non-cropped) and cropped images trained with ResNeXt50. The last convolution layer’s coarse localization map (before AdaptiveAvgPool2d) reflects the important regions in the input image to detect a particular type of COD. The obtained Grad-CAM is normalized and resized to the original image size. A mask image is generated from Grad-CAM with a threshold of 100. The contours are drawn using the mask image and visualized on the input fundus image as shown in Fig. 5 (iv & viii). Contours are indicated in green when the prediction score is greater than or equal to 0.5; otherwise, they are highlighted in red. Owing to the difficulties in identifying minute lesions (e.g., microaneurysms, drusens, cup to disc ratio, etc.), DL models trained on non-cropped images failed to detect a majority of early-stage ocular diseases (refer Fig. 5 ii-iv). In contrast, the proposed approach performed well in accurately identifying the majority of lesions, thus aiding in the generation of explainable predictions.

The proposed ensemble model outperformed several state-of-the-art models [51, 54, 55] in terms of Kappa and *F*_1_ scores. AUC is insensitive to class imbalance, i.e., when the labels include many zeros, correctly detecting them may also lead to high AUC. As a result, a high *F*_1_ score is more significant than a high AUC in cases with a high class imbalance. For the studies using ODIR training using 1166 patients’ data, the models put forth by He et al. [57] and Li et al. [56] showed better results. Due to the lack of precise patient IDs for each split, our proposed method could not be evaluated and compared to these models. Additionally, owing to the fusion of features, obtaining evidence for the output predictions is difficult with these models. With the proposed method, the evidence can be visualized for each predicted label (true or false). Moreover, He et al. [57] utilized diagnostic keywords indicative of actual diagnosis along with fundus images to augment their accuracy. Due to this, there is a dependency on expert generated diagnosis reports, which imposes an additional load on the learning models.

All other preprocessing strategies, except for the RoI cropping method, had no significant impact on the prediction performance of DL models (please see Table 5). Though the images created with CLAHE and the MIRNET seemed to improve contrast visually, the techniques did not significantly boost the CNN performance. The efficiency is similar to that of the RGB channels when only the green channel is used, but the number of training parameters is decreased by around 10000. Hence, this helps with efficient training and inference. Furthermore, the vessel segmentation technique that was primarily investigated for DR detection [35–37], had no discernible effect on COD detection. Table 9 summarises the advantages, disadvantages, and execution time (in milliseconds) of the various experimental preprocessing techniques using fundus images. Table 9 shows the average time taken by all preprocessing methods for five random images when executed on Apple M1 CPU processor with 16GB RAM. It can be observed that certain preprocessing techniques enhance brighter structures/lesions while others emphasise darker structures or lesions in fundus images. Thus, using a combination of preprocessing techniques improves performance.

**Table 9:** Summary of preprocessing techniques for COD detection using fundus images

Method	Observed execution time (in *ms*)	Observations on COD fundus images
Individual channels	0.0348	Green channel best differentiates blood vessels, exudates, and haemorrhages and is often used to identify DR. Unlike the red and blue channels, this channel is neither under- nor over-illuminated. CNN trained only on green channels needs fewer training parameters. However, the green channel has less information on the optic disc, which is necessary for diagnosing other eye illnesses such as glaucoma. The red channel is the brightest, and it can distinguish the optic disc from other portions of the fundus image. Segmentation of the optic disc is primarily used to identify eye disorders such as glaucoma. However, it is more noisy, so it is not suitable for detecting other COD. The blue channel is the darkest component and has not been extensively studied for use in detecting COD.
CLAHE	2.855	It is a sharpening filter that increases the contrast of fundus images and is commonly used to detect DR and glaucoma. Enhances the low-contrast regions, especially the contrast enhancement of microaneurysms and small blood vessels. However, if the majority of the pixels in the fundus image are dark, an excessive enhancing effect may occur, distorting the image’s overall visibility.
Gaussian convolution	2.259	The Gaussian smoothed image reduces noise, and when subtracted, the fundus image is sharpened. This increases the contrast between blood vessels and the surrounding environment and is often employed in DR detection. However, minute features are obscured in brighter regions, such as the optic disc. Additionally, border areas for the brighter photos exhibit additional artefacts.
MSR	17.667	The difference between the input value (centre) and normalized surround or neighbourhood values determines MSR output. The MSR technique enhances images captured under a variety of nonlinear lighting conditions to the degree that a person would perceive them in real time. However, several parameters in this improvement procedure are image-dependent and must be modified accordingly. Additionally, the algorithm will introduce extra artefacts into the enhanced image for the regions with significant brightness changes.
MIRNET	60896.423	Full-resolution processing recovers the original image’s high-quality content from its degraded counterpart, while the complementary set of parallel branches gives enhanced contextual features. MIRNET establishes links between features both inside and across branches of varying sizes. The method of feature fusion enables dynamic adaptation of the receptive field without jeopardizing the original feature details. However, additional artefacts are seen in images with a high number of brighter lesions.
Vessel segmentation	6056.545	Segmentation of the vascular structure is commonly utilized to detect COD such as AMD, diabetic retinopathy, and glaucoma. However, segmented vessels often have poor contrast, particularly thin and tiny vessels. Identifying minute changes in vascular structure for the purpose of detecting COD is often challenging without patient demographic information. Other retinal structures (optic disc, macula, fovea, etc.) and lesions (microaneurysms, exudates, etc.) also contribute significantly to the detection of COD.
Vessel inpainting	6133.436	Blood vessel inpainting is a technique that includes inpainting segmented vessels with a fundus backdrop. It is primarily used to diagnose glaucoma by the localization and segmentation of the optic disc. However, the anatomy of the vasculature is critical in diagnosing other COD.

Condition-level augmentation improved prediction accuracy for the *Normal*, *DR*, *Cataract*, *AMD*, and *Myopia* classes. It did not, however, improve prediction for *Glaucoma* or *Other* categories of diseases. We believe this limitation could be addressed by including more representative images for these classes (particularly with minute lesions). To further investigate the impact of augmentation on prediction performance, the highest-scoring DL model (ResNeXt) trained on ODIR is tested on the publicly available DDR test dataset [83]. The DDR dataset makes use of the International DR Grade Classification [84] (ranging from 0 to 4), as well as a special label (5) for low-quality images. These ungradable images are excluded from our study due to their low quality. The remaining dataset (3,759 images) is split into normal (0) and abnormal (1,2,3,4). The foreground region is cropped using the proposed method (refer Section 3) and tested using the ODIR pretrained ResNeXt model. The final score is obtained using the predictions of the “*Normal*” class. The results of the three augmentation techniques for DR screening are presented in Table 10. The observations revealed that the condition-level augmented model holds promise for building a generalizable DL model. The inference for DR screening is also achieved using an ensemble of models trained on the ODIR dataset. The proposed ensemble model outperformed a patch-based lesion localization deep network proposed by [85] in terms of AUC and sensitivity scores (refer Table 10).

**Table 10:** Comparative evaluation of augmentation and preprocessing techniques on DDR testset

Method	Observed performance			
	Kappa	*F*_1_	AUC	Sensitivity
No augmentation	0.5797	0.7898	0.8669	0.9276
Batch-level augmentation	0.5812	0.7906	0.8793	0.9170
Condition-level augmentation	0.6052	0.8026	0.8769	0.9091
Ensemble 1 *(ResNeXt50, EfficientNetB7 & VGG16)*	0.6196	0.8098	0.8749	0.9563
Ensemble 2 *(RoI cropped, green channel & MSR)*	0.6302	0.8151	0.8730	0.9570
Patch-based lesion localization model [85]	–	–	0.8480	0.8910

### Pilot study

A pilot study was conducted to understand the contribution of each preprocessing technique, to assess their effectiveness in aiding ophthalmologists to make better clinical decisions. The study was designed based on the ODIR test dataset, and the objective was to evaluate the most suitable approach in real-world scenarios when trained human experts diagnose COD. Two specialist ophthalmologists were provided with the ODIR test image collection for this purpose. Two different experiments were carried out by separately providing them with the *RoI cropped color*, and *RoI cropped green channel* images. The operational definitions used by trained experts to annotate the images are listed in Table 11. Any artefacts in the fundus images, like mild haze and rim defects, were ignored. In some of the images, the optic disc was not captured properly during fundus photography, which posed difficulties in interpretation. Diagnosis in the absence of clinical history and patient demographics also added to the challenges. The observations reported by medical experts with reference to the patient-level evaluations carried out by them were considered, and the observed performance was evaluated using the Kappa score, AUC and *F*_1_ score. The results of this pilot study are tabulated in Table 12. It can be noted from the table that the *RoI cropped color* images are much more effective compared to the *RoI cropped green channel* images. It can be noted from the tabulated results that the R*OI cropped color* images are much more effective when compared to the *RoI cropped green channel* images. This is consistent with the results obtained with the CNN model during our experimental evaluation (refer Table 5).

**Table 11:** Operational definitions used by domain experts for the testset image labeling

Ocular disease	Operational definition
Cataract	Fundus image is hazy, may not permit or only permits a faint view of the disc, macula and the vascular arcades.
Diabetic retinopathy	Fundus image shows evidence of microaneurysms with one or more of the following: dot and blot hemorrhages, intraretinal microvascular abnormalities (IRMA), hard exudates, venous beading, neovascularization.
Hypertensive retinopathy	Fundus image shows evidence of arteriolar narrowing and arteriovenous crossing changes with any of the following changes: flame-shaped hemorrhages, soft exudates, hard exudates, optic disc edema.
Glaucoma	Fundus image shows cup:disc ratio of > 0.5 with nasalization of vessels.
AMD	Fundus image shows evidence of soft or hard drusen with pigmentary changes in the macula.
Myopia	Fundus image shows evidence of a large temporal or an annular crescent with chorioretinal degenerative changes.
Others	Fundus image shows other fundus lesions like medullated nerve fibers, macular hole, pigmentation, or any other lesion unrelated to the above conditions.
Normal	Fundus image shows a normal disc and macula, without any of the above possible diagnosis.

**Table 12:** Results of pilot study -illustrating its benefits to the ophthalmologists in their diagnosis

Input images	Observed performance		
	Kappa	AUC	*F*_1_
RoI cropped images	0.2631	0.6382	0.8078
Green channel	0.2387	0.6291	0.7958

## Conclusion and future work

Early diagnosis of COD is important for clinical decision-making and can potentially eliminate vision impairment. However, existing manual screening approaches are cumbersome and time-consuming. In this paper, we presented a comprehensive study on the effectiveness of preprocessing techniques for automated COD diagnosis. Experiments revealed that ResNeXt was most effective at modelling the very imbalanced and noisy ODIR dataset, when compared to the other state-of-the-art transfer learning approaches considered for the evaluation. We demonstrated that the models trained on images processed using the proposed RoI Segmentation Algorithm outperformed those models trained on original non-cropped input images by a significant margin. The interpretability was demonstrated using the CNN learned features, thereby establishing the impact of the proposed RoI segmentation on instigating trust in intelligent healthcare systems. The experimental results show that, except for the RoI segmentation method, the other preprocessing strategies do not impact much on CNN performance. The proposed ensemble approach with batch-level augmentation was found to be superior when compared to state-of-the-art techniques benchmarked on the ODIR-5k dataset. As part of extended work, we aim to augment the model and approaches presented to accommodate a detailed study of the impact of attention layers at various stages of inference using CNNs. Recently, image enhancement approaches based on optimization algorithms [86–90], have been proposed that have the potential to improve CNN performance; these methods will be experimented with as part of future work. Additionally, we intend to address the high class imbalance problem and plan to explore the possibility of using patient profiling via automated generation of textual findings while considering both eye conditions.

## Footnotes

1 Submitted to: https://odir2019.grand-challenge.org/evaluation/challenge/submissions/create/
